# Hydrocephalus and arthrogryposis in an immunocompetent mouse model of ZIKA teratogeny: A developmental study

**DOI:** 10.1371/journal.pntd.0005363

**Published:** 2017-02-23

**Authors:** Jose Xavier-Neto, Murilo Carvalho, Bruno dos Santos Pascoalino, Alisson Campos Cardoso, Ângela Maria Sousa Costa, Ana Helena Macedo Pereira, Luana Nunes Santos, Ângela Saito, Rafael Elias Marques, Juliana Helena Costa Smetana, Silvio Roberto Consonni, Carla Bandeira, Vivian Vasconcelos Costa, Marcio Chaim Bajgelman, Paulo Sérgio Lopes de Oliveira, Marli Tenorio Cordeiro, Laura Helena Vega Gonzales Gil, Bianca Alves Pauletti, Daniela Campos Granato, Adriana Franco Paes Leme, Lucio Freitas-Junior, Carolina Borsoi Moraes Holanda de Freitas, Mauro Martins Teixeira, Estela Bevilacqua, Kleber Franchini

**Affiliations:** 1 Brazilian Biosciences National Laboratory, LNBio, Rua Giuseppe Máximo Scolfaro, 10.000, Polo II de Alta Tecnologia de Campinas, Campinas, SP, Brazil; 2 Department of Cell and Developmental Biology, Institute of Biomedical Sciences, University of São Paulo, São Paulo, SP, Brazil; 3 Laboratório de Imunofarmacologia, Departamento de Bioquímica e Imunologia, Instituto de Ciências Biológicas, Universidade Federal de Minas Gerais, Avenida Antônio Carlos, 6627, Belo Horizonte, MG, Brazil; 4 CPqAM-Fiocruz. Federal University of Pernambuco, Av. Professor Moraes Rego s/n, Cidade Universitária, Recife, PE, Brazil; George Mason University, UNITED STATES

## Abstract

The teratogenic mechanisms triggered by ZIKV are still obscure due to the lack of a suitable animal model. Here we present a mouse model of developmental disruption induced by ZIKV hematogenic infection. The model utilizes immunocompetent animals from wild-type FVB/NJ and C57BL/6J strains, providing a better analogy to the human condition than approaches involving immunodeficient, genetically modified animals, or direct ZIKV injection into the brain. When injected via the jugular vein into the blood of pregnant females harboring conceptuses from early gastrulation to organogenesis stages, akin to the human second and fifth week of pregnancy, ZIKV infects maternal tissues, placentas and embryos/fetuses. Early exposure to ZIKV at developmental day 5 (second week in humans) produced complex manifestations of anterior and posterior dysraphia and hydrocephalus, as well as severe malformations and delayed development in 10.5 days *post-coitum* (dpc) embryos. Exposure to the virus at 7.5–9.5 dpc induces intra-amniotic hemorrhage, widespread edema, and vascular rarefaction, often prominent in the cephalic region. At these stages, most affected embryos/fetuses displayed gross malformations and/or intrauterine growth restriction (IUGR), rather than isolated microcephaly. Disrupted conceptuses failed to achieve normal developmental landmarks and died *in utero*. Importantly, this is the only model so far to display dysraphia and hydrocephalus, the harbinger of microcephaly in humans, as well as arthrogryposis, a set of abnormal joint postures observed in the human setting. Late exposure to ZIKV at 12.5 dpc failed to produce noticeable malformations. We have thus characterized a developmental window of opportunity for ZIKV-induced teratogenesis encompassing early gastrulation, neurulation and early organogenesis stages. This should not, however, be interpreted as evidence for any safe developmental windows for ZIKV exposure. Late developmental abnormalities correlated with damage to the placenta, particularly to the labyrinthine layer, suggesting that circulatory changes are integral to the altered phenotypes.

## Author summary

Previously thought to produce a harmless bout of fever associated with skin rash and muscle, or joint pain, the Zika virus (ZIKV) is an important cause of morbidity/mortality to human embryos/fetuses. Different from other models, here we report data from wild-type immunocompetent mice, rather than transgenic animals with suppressed immune responses. We found that intravascular ZIKV produced infection of maternal tissues, placentas and conceptuses, but that embryos/fetuses were comparatively much more affected than pregnant females, which seemed to tolerate well the viral challenge with no signs of encephalopathy. Importantly, 10.5 days *post-coitum* (dpc) embryos exposed to ZIKV at embryonic day 5 (second week in humans) displayed dysraphia, which is a regional failure of neural tube closure, and hydrocephalus, which is a symptom recently shown to precede microcephaly in humans. Characteristic phenotypes in more developed embryos/fetuses included abnormal articular postures analogous to arthrogryposis, which are typical human congenital contractures, gross and generalized malformations and intra uterine growth restriction (IUGR), rather than isolated microcephaly. Some developmental abnormalities and IUGR correlated with placental damage, suggesting that loss of placental function may play an important role in the disease. We believe our model is an asset in the search for useful concepts and prospective therapies for ZIKV because it better reproduces the human condition.

## Introduction

Zika virus (ZIKV) is a mosquito-borne flavivirus that was initially thought to produce a benign disease characterized by mild fever, muscle and joint pain, rash and conjunctivitis [1]. However, the recent ZIKV epidemic in Brazil has been associated with severe transient, as well as irreversible neurological manifestations, such as ascending paralysis (Guillain-Barré syndrome) and microcephaly, respectively [2–6]. Furthermore, it is becoming increasingly clear that ZIKV pathogenicity is not restricted to the aforementioned conditions [7]. In fact, ZIKV shows many similarities to ‘TORCH’ pathogens (*Toxoplasma gondii*, other, rubella virus, cytomegalovirus and herpes simplex virus), especially in the way it accesses embryos and fetuses [8], a realization that increases our public health concerns.

In order to face the challenges posed by an infectious agent with such potential, the scientific community worldwide directed its attention to the biology of ZIKV and its pathogenicity. However, at this junction, there are few clues on the mechanisms triggered by viral infection to damage the embryonic and/or fetal human. This is because we are still lacking a suitable animal model of intrauterine injury after exposure to ZIKV.

Large strides have been made towards a relevant animal model for ZIKV teratogeny [9–15]. However, the currently available models are still somewhat limited. This is because these models rely on unnatural prerequisites such as genetically modified immunodeficient animals, exaggerated viral loads and facilitated access of the virus to target tissues via surgical procedures (e.g. direct brain injection) [10,12,16]. For instance, although the model of Miner *et al*. [9] utilizes viral loads and infection routes eminently compatible with the epidemiological setting, it depends largely on the failure of the host to mount an interferon-based immunological response. Exposure to ZIKV in this setting compromises the conceptus, but also induces maternal encephalitis, which has not been shown to be a necessary component, or frequent association with ZIKV teratogeny in humans [1,17]. Although the use of IFN1 antibodies can eliminate encephalitis from their model [9], the associated costs may be prohibitive for most laboratories. Despite its elevated viral load, the model described in Cugola *et al*. [11] is not capable of infecting immunocompetent wild-type C57 mice, which is an important issue to be clarified, as contradictory results have been published [13]. More recently, Yockey and colleagues [13] described an intriguing model of ZIKV infection through intravaginal exposure that greatly expands our understanding of ZIKV pathogeny. However, even as Yockey *et al*. [13] report brain infection in conceptuses from immunocompetent dams, their model is not associated with overt morphological damage to the embryonic/fetal brain in wild-type animals. Therefore, our assessment is that we need models of ZIKV teratogeny that are better related to the human disease. Here we present a mouse model of developmental damage induced by direct maternal injection of ZIKV into the external jugular vein. Our results established that immunocompetent animals from widely available wild-type FVB/N and C57BL/6J strains are indeed affected by ZIKV, which produces a host of congenital abnormalities including dysraphia, hydrocephalus, arthrogryposis, gross malformations and disturbances such as intrauterine growth restriction (IUGR), which is often correlated with damage to the placenta.

## Methods

### Virus isolate, cell culture and viral propagation

The ZIKV HS-2015-BA-01 was isolated from a serum sample of a ZIKA infected patient during a 2015 outbreak in Bahia State, Brazil. The complete cds (polyprotein gene) sequence of the isolate is available under GenBank accession KX520666.1. ZIKV HS-2015-BA-01 was isolated by passage in C6/36 mosquito cells, up to passage 3. Aliquots from cell culture contents were repeatedly processed for virus detection through reverse transcription polymerase chain reaction (RT-PCR). Subsequently, the virus was propagated and titrated in the green monkey kidney epithelial cell line Vero using the method described by Medina et al. [18]. Vero cells were cultivated in DMEM (Sigma Aldrich) supplemented with 10% fetal bovine serum, 100 units/ mL of Penicillin and 100 μg/mL of Streptomycin, at 37°C, 5% CO_2_.

### Detection of ZIKV infected cells by immunofluorescence

ZIKV infected Vero cells were fixed with paraformaldehyde 4% (w/v) for 30 min at room temperature, treated with 0.25% (v/v) Triton-X for 30 min and incubated with the primary monoclonal antibody 4G2 from mouse ascites (raised against the flaviviral envelope protein) in Phosphate-Buffered Saline (PBS) 1X containing 2.5% FBS at 37°C for 2 h [19]. After two washes with PBS 1X, the cells were incubated with AlexaFluor594 conjugated goat anti-mouse IgG (Thermo Scientific) and 5 μg/mL of DAPI (4',6 diamidino-2-phenylindole) (Sigma Aldrich) in Dulbecco’s PBS at room temperature for 1 h and then washed again twice with PBS 1X. After the final washing cycle, digital images were acquired at 20X magnification using a high-throughput confocal fluorescence imaging system (Operetta Perkin Elmer).

### Mice

Adult FVB/NJ (JAX#1800) and C57BL/6J (JAX#664) mice were housed in the pathogen-free animal facility at the Laboratory of Genome Modification (LNBio). Animals were maintained on a photoperiod of 12:12 light/dark cycle at 21–24°C. Landmarks typical of relevant developmental days were compiled from Kauffman [20].

### Intravascular ZIKV infection

Six to eight-week-old pregnant mouse females (5.5, 7.5, or 9.5 dpc) weighing 22–28 g were anaesthetized with intraperitoneal injections of ketamine/xylazine (100/10 mg.kg^-1^). Since more traditional infections routes, such as lateral tail vein injection (n = 3 at 5.5 dpc) and intraperitoneal injection (n = 8 at 5.5 dpc) had no impact in embryos, a jugular venous access was established as previously described [21]. Briefly, after positioning the animal on its back, we performed midscapular and diagonal incisions, isolated the right external jugular vein and inserted a polyethylene cannula (PE-10) into it. A viral stock solution of 10^9^ plaque-forming units per ml was serially diluted with PBS to produce 100 μl aliquots containing viral loads of 10^8^, 10^7^_,_ 10^6^, 10^5^, 10^4^, 10^3^ plaque-forming units. These viral loads were administered using a 1 ml plastic syringe with a polished 20-gauge needle connected to the PE-10 cannula. Control pregnant females were injected with 100 μl of PBS. After jugular infection, the PE-10 cannula was withdrawn, the jugular vein closed, and the animals sutured and allowed to recover under heating from an infrared lamp. ZIKV-injected pregnant females and PBS-injected controls were monitored daily after infection.

### Morphometry

Individual embryos, fetuses and placentas from ZIKV-infected pregnant females, as well as their corresponding PBS-injected controls and reference animals from our mouse colony were collected from 9.5 dpc to 18.5 dpc (S1 Table). Optical images were captured before and after amnion dissection, using a Nikon stereomicroscope. Immediately after dissection, embryos, fetuses and their respective placentas were weighed and their crown to rump lengths were measured using a digital caliper and a previously calibrated ImageJ software (https://imagej.nih.gov/ij/). Brain size was evaluated by measuring both biparietal and occipital-frontal diameters using ImageJ software. Linear regression and One-way ANOVA followed by Tukey’s multiple comparisons test were performed using GraphPad Prism version 7.0a for Mac OS X (GraphPad Software, La Jolla California USA, www.graphpad.com).

### Mouse developmental staging

The ontogenetic periods associated with each conceptus were determined according to the staging scheme of Theiler [22], complemented by Downs and Davies [23], as well as by Kauffman [20]. To determine the staging at the presumed time of death, we utilized the status of both fore and hindlimbs, as well as additional characters such as: 1- extent of covering of the external acoustic meatus by the pinna of the ear; 2- degree of eyelid closure; presence, or absence of skin wrinkles in the neck, trunk and limbs; 3- status of the vibrissae (arrangement of rows and eruption); 4-presence, or absence of the sinus hair follicle; 5- status of the umbilical hernia. A state of delayed development at the time of death was inferred whenever a given conceptus failed to present the complete set of morphological characters typical of its estimated stage at the time of death.

### Histology and immunohistochemistry

Embryos, fetuses and their respective placentas, as well as the spleens from ZIKV-infected pregnant females were collected at 10.5, 12.5, 16.5 and 18.5 dpc. After fixing overnight in 4% paraformaldehyde, the tissues were embedded in paraffin blocks and cut into 6 μm-thick sagittal sections using a Leica microtome. Embryos were divided in two sagittal halves and both fetal and maternal sides of placentas were included. Embryos at 18.5 dpc were decalcified before embedding and stained for Hematoxylin & Eosin according to established protocols. For immunoreactions, dewaxed and hydrated placental sections were sequentially incubated in: 1- three washes with 2% H_2_O_2_ in Tris-buffered saline (TBS) for 5 min to quench endogenous peroxidase activity; 2- blocking solution containing bovine serum albumin 2% (v/v) in TBS and primary antibodies for 1 h at room temperature; 3- peroxidase-conjugated goat anti-rabbit IgG secondary antibody (1:200 in TBS, Abcam #6721) at 37°C for 1 h, followed by development with DAB (Sigma-Aldrich) and counterstained with Mayer's hematoxylin. Primary antibodies were rabbit polyclonal anti-CD31 (Abcam#28364), anti-SCL16A3 (Abcam #1904987) and anti-EpCAM (Abcam #71916) respectively diluted 1:50, 1:150 and 1:100 in TBS (v/v). For ZIKV (flaviviral) staining, a blocking solution with goat IgG polyclonal isotype control (1:200 in TBS, Abcam #37373) was utilized for 30 min at room temperature. This was followed by unconjugated rabbit F(ab) fragment anti-mouse IgG (1:200 in TBS, Sigma #3700999) for 1 h at room temperature and then by mouse flavivirus-specific monoclonal IgG 4G2 (hybridoma D1-4G2-4-15, ATCC HB-112) (1:200 in TBS) for 2 h at 37°C. Peroxidase-conjugated goat anti-mouse IgG (1:200 in TBS, Abcam, #6789) was used as secondary antibody at 37°C for 1 h, followed by development with DAB and counterstaining with Mayer's hematoxylin. Negative controls were carried out by omitting step (4) of the immunohistochemical reaction.

### Cloning and sequencing of a ZIKV-derived amplicon

A 339 bp ZIKV amplicon (1791–2130) was amplified by PCR with the following set of primers: forward 5’- GATAAACTTAGATTGAAGGGCGTG -3’; reverse 5’- TCCAATGGTGCTGCCACTC -3’, using a cDNA template obtained from ZIKV-infected Vero cells. The amplicon was cloned into pGEM-T vector (PROMEGA) following manufacturer's instructions. The purified pGEM-ZIKV plasmid was sequenced to verify ZIKV viral identity and used to derive standard curves in real time PCR assays.

### Quantitative real time PCR

Quantitative real time PCR (qRT-PCR) was utilized to confirm maternal ZIKV infection. Samples were homogenized in Trizol Reagent (Invitrogen), and total RNA was isolated. The isolated RNA (2 μg) was used for cDNA synthesis with the Superscript pre amplification system, following manufacturer's instructions. The qRT-PCR was performed using a MX3000P system (Stratagene, La Jolla, CA) and SYBR green reagent (Applied Biosystem–USA). We designed qRT-PCR primers to amplify a 148bp amplicon (1808–1955). Primer sequences are forward 5’-AGGGCGTGTCATACTCCTTG-3’ and reverse 5’-TGCATGTCCACCGCCATCT-3’. Each qRT-PCR contained 30 ng of reverse-transcribed RNA, each primer at 400 nM, and 6 μl of SYBR Green PCR Master Mix (Invitrogen) in a final volume of 12 μl. Each sample was analyzed in triplicate. PCR conditions were: 50°C for 2 minutes (1 cycle); 95°C for 5 minutes (1 cycle); 95°C for 30 seconds, 59°C for 45 seconds and 72°C for 45 seconds (35 cycles). Since pGEM-ZIKV also harbors this amplicon, this plasmid was used to generate the standard curve, which allowed determining virus copy number in the tissue samples. Samples were run in triplicate. The virus copy number was interpolated from the cycle thresholds of SYBR green qRT-PCR assay, using standard linear curve (R^2^ values of 0.99) generated from known amounts of control pGEM-ZIKV plasmid (range of 1 x 10^7^ to 1 x 10^1^ copies/reaction), as described previously [24,25].

### Viral load assessment in samples

Blood and organ samples from ZIKV-infected and PBS-injected pregnant mice were stored at -80°C until viral assessment. To measure ZIKV viremia, 50–100 μl of blood were collected through the lateral tail vein at indicated time points after infection. Samples were thawed and submitted to a tissue culture infectious dose (TCID_50_) assay in Vero cells. Briefly, whole blood, or minced suspensions of organs were serially diluted and placed in cell culture plates containing confluent Vero cell monolayers for one hour. Samples were removed and fresh complete DMEM was added to the cell plates. Cell culture plates were maintained in the incubator for 5 days, following fixation with 10% w/v formaldehyde and staining with methylene blue at 1% w/v. TCID_50_ was calculated by direct observation of cell culture plates.

### Western blotting

Individual placentas and maternal tissues (brain, kidney, spleen and liver) from control, or ZIKV-infected pregnant females were harvested at 12.5 dpc, minced and lysed in ice-cold RIPA buffer (50 mM Tris-HCl pH 8.0, 150 mM NaCl, 0.5% sodium deoxycholate, 0.1% SDS, 1% Nonidet P40) supplemented with Complete Protease Inhibitors (Roche), DNAse I (Promega) and RNAse A (Sigma). Subsequently, the lysate was cleared at 14.000×g at 4°C for 30 min and protein concentration was determined using the BCA Protein Assay Reagent Kit (Thermo Scientific). Fifty micrograms (50 μg) of proteins from the extract were mixed to Laemmli buffer containing beta-mercaptoethanol, loaded into a gel and submitted to 10% SDS-PAGE electrophoresis and subsequently transferred to nitrocellulose membrane. After blocking with 5% skim milk in Tris-buffered saline (25 mM Tris, pH 7.4, 137 mM sodium chloride, 2.7 mM potassium chloride) containing 0.05% Tween 20 (TBST) for 1 h, membranes were hybridized with the flavivirus-specific monoclonal antibody 4G2 (1:1000 in TBST, hybridoma D1-4G2-4-15, ATCC HB-112) for 1 h. After washing with TBST, membranes were incubated with the goat anti-mouse horseradish peroxidase-conjugated secondary antibody for 1 h and washed with TBST. Detection was performed using Pierce™ ECL Western Blotting Substrate (Thermo Scientific) according to the manufacturer's recommendations.

### Ethics statements

This study was carried out in strict accordance with the recommendations set forth in the Guide for the Care and Use of Laboratory Animals of the Brazilian National Council of Animal Experimentation (http://www.cobea.org.br/) and the Federal Law 11.794 (October 8, 2008). The Institutional Committee for Animal Ethics of the Brazilian Center for Research in Energy and Materials (CEUA-CNPEM, License 29-B) approved all the procedures used in this study.

## Results

### A wild-type (FVB/NJ and C57BL/6J) immunocompetent model of ZIKV infection in mice

**Fig 1:**
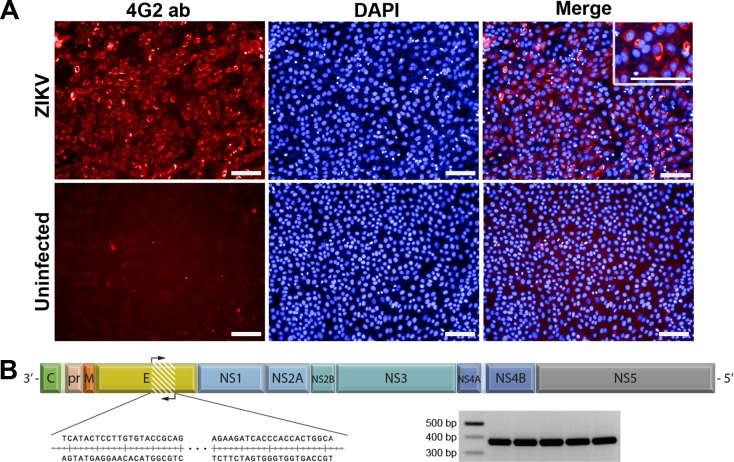
ZIKV *in vitro* infection and genomics. (**A**) Vero cells were infected with ZIKV for 72 h and submitted to indirect immunofluorescence with the anti-flaviviral 4G2 monoclonal antibody. Inset: higher magnification of 4G2 staining surrounding the nuclei of infected Vero cells. Red: Zika virus labeling; Blue: Cell nuclei stained with DAPI; scale bars, 0.1 mm. (**B**) The linear ssRNA (+) (10,640 bp) complete cds (+ partial UTRs) sequence of ZIKV (HS-2015-BA-01), highlighting the 339 bp amplicon of the E protein gene (see DNA bands from five independent samples in the inset), which was cloned and sequenced.

To characterize the ZIKV strain used in our experiments, we infected Vero cell cultures with aliquots of our working ZIKV stocks. Monolayers of Vero cells were stained with an anti-flavivirus E protein antibody (4G2), which is commonly used in *in vitro* assays of flavivirus infection. As shown in Fig 1A, ZIKV HS-2015-BA-01 efficiently infected Vero cell monolayers, which were almost completely positive for the 4G2 antigen (red). Uninfected cell cultures were negative for flavivirus E-protein staining. As the 4G2 antibody detects a flaviviral E protein epitope, we further confirmed the identity of ZIKV by sequencing an amplicon of the ZIKV E-protein gene, which established it as a *bona fide* fragment of the ZIKV genome. These results were complemented by the complete cds (polyprotein gene) sequencing of the ZIKV HS-2015-BA-01 strain (Fig 1B), which is available in GenBank under the accession code KX520666.1.

**Fig 2:**
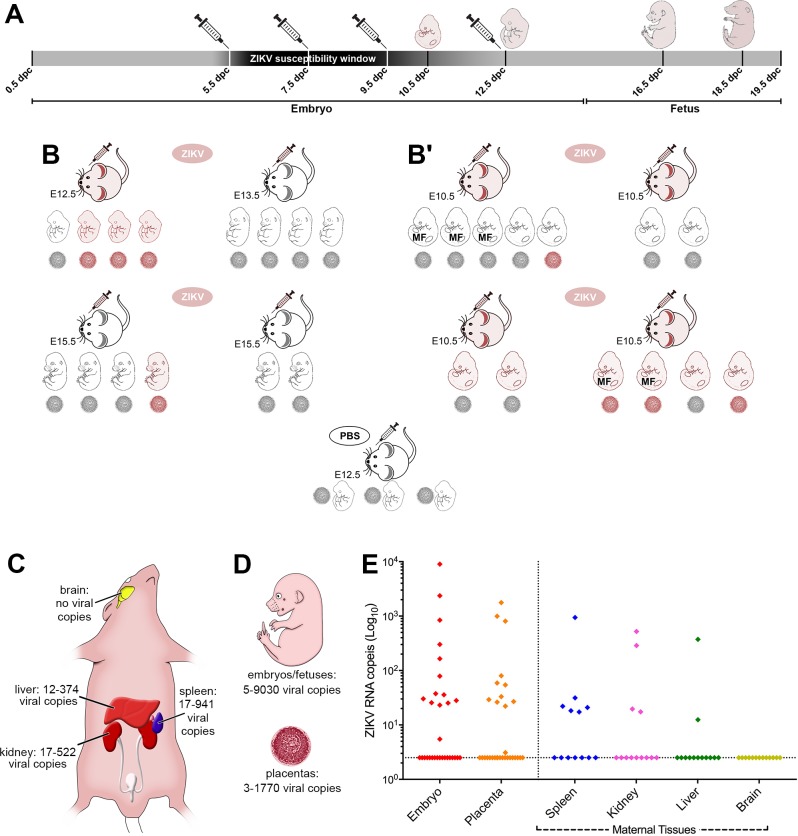
Window of susceptibility in a wild-type (FVB/NJ and C57BL/6J), immunocompetent model of ZIKV infection in mice. (**A**) Scheme of intravascular injection via the external jugular vein of 10^5^ plaque-forming units (pfu). ZIKV produces a host of morphological disruptions at days 5.5, 7.5 and 9.5 days, but not at 12.5 days *post coitum* (dpc). This outlines a window of sensitivity to ZIKV teratogeny in mice spanning late gastrulation to late neurulation. Schematic syringes and embryos indicate injection and harvest days, respectively. (**B-B'**) Scheme representing maternal, fetal and placental outcomes after intravascular injection of ZIKV into pregnant females at 5.5 dpc (see Table 1), as determined by quantitative real time PCR (qRT-PCR). ZIKV mRNA detection is graphically represented by pink filling and the developmental stage at time of harvest is indicated for every litter (in dpc). Affected litters in **B** were all dead at the time of harvest and those affected in **B'** were alive. MF indicates malformed embryos. (**C-D**) Schematic representation of the ZIKV burden in pregnant females, embryos/fetuses and placentas as indicated by the number of viral copies detected by qRT-PCR. (**E**) qRT-PCR results from embryos (n = 32, 14 ZIKV positive), placentas (n = 32, 12 ZIKV positive), maternal spleen (n = 13, 6 ZIKV positive), kidney (n = 13, 4 ZIKV positive), liver (n = 13, 2 ZIKV positive), and brain (n = 13, no ZIKV positive).

The rationale for our model is underpinned by the conjecture that adult immunocompetent wild-type mice are resistant to ZIKV infection, after cutaneous exposure due to defenses that are at play in the skin, subcutaneous tissues and lymph nodes, as shown for the Dengue virus [26]. Thus, we hypothesized that ZIKV inoculation directly to the circulation would allow ZIKV infection of maternal target tissues and placenta. Accordingly, we devised a scheme in which we infected pregnant FVB/N and C57BL/6J females with ZIKV via an external jugular access (Fig 2A).

The establishment of comprehensive relationships between inoculum size and developmental damage is outside of the scope of this initial model characterization. However, we established that viral loads of 10^3^ to 10^4^ plaque-forming units (pfu) either produced no phenotypes, or were associated with very low frequencies of developmental abnormalities, which precluded their practical use. On the other hand, viral loads of 10^6^, 10^7^ and 10^8^ were too aggressive, producing too many early embryonic resorptions (i.e. dead embryonic sacs compatible with 5.5 to 7.5 dpc stages), which made them impractical to model the effects of ZIKV on late embryonic morphogenesis, or fetal maturation. Accordingly, in pilot experiments, we obtained the best results when pregnant FVB/NJ or C57BL/6J females were injected with 10^5^ pfu in PBS (see methods). The proportion of conceptuses undergoing early resorptions associated with the ZIKV 10^5^ pfu inoculum was 8.45% (06/71), which is not significantly different from 10.26% (4/39) of resorptions in PBS controls (*p* = 0.7408, *Chi*-square test). This is consistent with our interpretation that the chosen viral load of 10^5^ pfu is ideal to investigate the late embryonic and fetal effects of ZIKV and, thereafter, we refer only to this viral titer. Pregnant females injected intravascularly with ZIKV recovered quickly from anesthesia and did not demonstrate signs of encephalopathy, or overall distress. Tables 1 and 2 provide a complete quantitative description of experimental and control animals utilized, viral titers, developmental periods, affected embryos and fetuses as well as other relevant parameters. The S1 Table lists all embryos and fetuses obtained from healthy, non-disturbed, pregnant females at our animal facility (reference embryos and fetuses).

**Table 1:** ZIKV (10^5^ plaque-forming units)-injected pregnant mouse dams and litters.

				Live embryos at harvest		Dead embryos at harvest				
Mouse strain	Injection (dpc)	Harvest (dpc)	Total embryos	Normal	Malformed	Outwardly normal	Malformed	Reabsorptions	Total dead	Ref.
C57BL/6J	5.5	9.5	11	10	0	1	0	0	1	
C57BL/6J	5.5	9.5	9	9	0	0	0	0	0	
C57BL/6J	5.5	11.5	10	7	0	0	1	2	3	
C57BL/6J	5.5	11.5	13	13	0	0	0	0	0	
C57BL/6J	5.5	11.5	7	7	0	0	0	0	0	
C57BL/6J	5.5	11.5	10	10	0	0	0	0	0	
C57BL/6J	5.5	12.5	10	9	0	0	1	0	1	Fig 6A and 6B
C57BL/6J	5.5	13.5	9	7	0	0	0	2	2	
C57BL/6J	7.5	18.5	8	7	0	0	1	0	1	Fig 6D and 6E
C57BL/6J	7.5	18.5	9	9	0	0	0	0	0	
FVB/NJ	5.5	10.5	11	8	3	0	0	0	0	Fig 5A and 5D
FVB/NJ	5.5	10.5	12	11	0	0	0	1	1	
FVB/NJ	5.5	10.5	12	12	0	0	0	0	0	
FVB/NJ	5.5	10.5	13	11	2	0	0	0	0	Fig 5B and 5C
FVB/NJ	5.5	10.5	11	10	0	0	0	1	1	
FVB/NJ	5.5	10.5	10	9	0	0	1	0	1	Fig 5E and 5E’
FVB/NJ	5.5	10.5	9	6	0	0	2	1	3	
FVB/NJ	5.5	11.5	10	9	0	0	0	1	1	
FVB/NJ	5.5	11.5	10	9	0	0	1	0	1	
FVB/NJ	5.5	11.5	9	0	0	0	2	0	2	
FVB/NJ	5.5	11.5	12	12	0	0	0	0	0	
FVB/NJ	5.5	14.5	13	12	0	0	0	1	1	
FVB/NJ	5.5	15.5	12	10	0	0	0	2	2	
FVB/NJ	5.5	15.5	12	10	0	0	0	2	2	
FVB/NJ	6.5	11.5	11	9	0	0	0	2	2	
FVB/NJ	9.5	16.5	13	0	0	5	8	0	13	Fig 7
FVB/NJ	12.5	16.5	10	9	0	0	0	1	1	
FVB/NJ	12.5	16.5	11	11	0	0	0	0	0	

**Table 2:** PBS-injected mouse dams and respective litters.

					Dead embryos at harvest				
Mouse strain	Injection (dpc)	Harvest (dpc)	Total embryos	Live embryos at harvest	Outwardly normal	Malformed	Reabsorptions	Total dead	Ref.
C57BL/6J	5.5	9.5	10	10	0	0	0	0	
C57BL/6J	5.5	11.5	13	13	0	0	0	0	
C57BL/6J	6.5	15.5	9	7	0	0	2	2	
C57BL/6J	7.5	18.5	11	10	0	0	1	1	Fig 6F
FVB/NJ	5.5	12.5	9	9	0	0	0	0	Fig 6C
FVB/NJ	7.5	16.5	10	9	0	0	1	1	
FVB/NJ	12.5	16.5	5	5	0	0	0	0	
FVB/NJ	12.5	16.5	11	10	0	0	1	1	

### ZIKV in pregnant mouse females

To establish whether intravascular ZIKV injections were effective to cause infection, females were injected at multiple stages of pregnancy, and tissue samples were collected for quantitative real time PCR assays (qRT-PCR) (Fig 2). As shown in Fig 2C–2E, we confirmed the presence of ZIKV in the spleen, liver and kidneys of 12.5 dpc pregnant females. In contrast, we did not detect copies of the ZIKV genome in any of the brain tissues examined.

**Fig 3:**
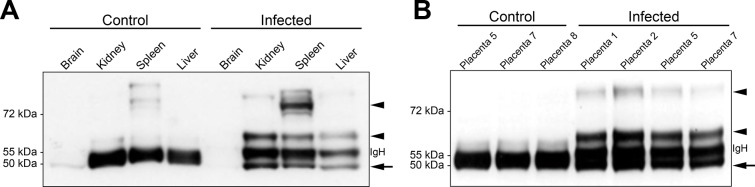
Detection of ZIKV E protein in an immunocompetent mouse model of ZIKV infection. Western Blot detection of flaviviral immunoreactivity in total protein extracted from maternal organs (**A**) and individual placentas (**B**) from pregnant females injected with ZIKV at 5.5 dpc and harvested at 12.5 dpc. Samples from ZIKV-infected tissues display a specific 50 kDa band, which corresponds to the mature flaviviral protein E (arrow). Higher immunoreactive bands represent immature polyprotein precursors such as prM/E (arrowheads). Note that no flaviviral protein was detected in the maternal brain. Nonspecific bands at 55 kDa observed in control samples (except the brain) correspond to tissue-derived immunoglobulin heavy chain (IgH).

To look for the presence of ZIKV protein in tissues, we analyzed brains, kidneys, spleens, and livers from control and infected females by Western blot. Using the anti-flavivirus 4G2 antibody, we observed specific bands in infected samples (Fig 3A and 3B), as previously described in reducing conditions [27,28], confirming ZIKV infection. The lower band (arrow in Fig 3A and 3B) corresponds to the mature viral protein E, which has a theoretical MW of 54.4 kDa, but migrates as a 50 kDa band, as described [29]. Higher MW bands are likely due to immature polyprotein precursors such as prM/E (precursor membrane/E protein) (arrowheads in Fig 3A and 3B). There was no evidence of ZIKV proteins in brain samples, in agreement with qRT-PCR findings (Fig 2E). Taken together, these data indicate that wild-type dams tolerate well intravascular ZIKV injection. When infected, pregnant females will harbor the virus in spleen, kidneys and, occasionally, in liver, but not in the brain, which is consistent with the absence of any neurological signs in these animals.

### A window of susceptibility to ZIKV-induced morphologic disruption

To outline a window of susceptibility for ZIKV-induced embryonic/fetal disruption, we set up ZIKV injections at 5.5; 6.5; 7.5; 9.5, as well as 12.5 days *post coitum* (dpc), and harvested embryos and/or fetuses at 9.5; 10.5; 11.5; 12.5; 13.5; 14.5; 15.5; 16.5 and 18.5 dpc (Fig 2A, Table 1). These experiments indicated that intravascular maternal injection of ZIKV is capable of producing embryonic, or fetal abnormalities from 5.5 to 9.5 dpc, but failed to produce noticeable malformations at day 12.5 dpc. These results suggest that exposure to ZIKV is comparatively far less likely to produce overt morphogenetic consequences at 12.5 dpc, than at earlier stages. Thus, there seems to be a developmental window of opportunity for ZIKV-induced teratogenesis that encompasses early gastrulation, neurulation and early organogenesis stages. It is important, however, to stress that our data should not be interpreted as evidence that there are any safe developmental stages when the conceptus is protected from ZIKV.

### The dynamics of ZIKV infection and developmental disruption

We next set out to determine the relationship between ZIKV exposure, maternal, placental, as well as conceptus infection and the presence, or absence, of malformations. To check whether ZIKV can reach the placenta and eventually the conceptus after maternal exposure and infection, we established a group of pregnant females that were injected at day 5.5 dpc with ZIKV (n = 4), or with PBS (control) (n = 1). In Fig 2B, we show that only one among the four ZIKV-injected females was infected at the day of harvest (12.5 dpc). Among the four pairs of embryos/placentas harvested from this 12.5 dpc ZIKV-infected pregnant female, three were found to display ZIKV infection, indicating vertical transmission, while the remaining embryo and its placenta were negative for the ZIKV genome. These data are in agreement with the documented human experience of vertical transmission from infected mothers and with the observation that not necessarily all conceptuses are infected by the virus following maternal exposure in twin pregnancies [17] (Fig 2B). The three other pregnant females exposed at 5.5 dpc and harvested at 13.5, or 15.5 dpc, tested negative for ZIKV, as did the PBS-injected control dam. Thus, at least at the times investigated (i.e. 13.5 and 15.5 dpc), our results are consistent with epidemiologic data in the human setting, in that not all exposed females are infected with the virus. Interestingly, but also disquietingly, we demonstrate that one fetus/placenta pair harbored copies of the ZIKV genome, in spite of the fact that the maternal organism tested negative for the viral genome at the day of harvest (15.5 dpc). This suggests that ZIKV may have reached this particular conceptus, but not its siblings, before the exposed pregnant female was able to mount a successful immunological response. None of the ZIKV-positive conceptuses in this group displayed overt morphological abnormalities.

Because ZIKV-infected conceptuses represented in Fig 2B did not display evident morphological changes, we repeated the injections at 5.5 dpc, but this time examined the embryos earlier, at 10.5 dpc, in the hope to find malformed embryos. In this dataset of thirteen living 10.5 dpc embryos, we found five malformed individuals (Fig 2B'). As represented in Fig 2B', we demonstrated the presence of ZIKV genome in two out of five malformed embryos. Unfortunately, we could not recover ZIKV RNA from the remaining three embryos, probably because of their very small sizes.

As depicted in Fig 2B, we confirmed that infected dams could transmit ZIKV vertically, but that the virus does not necessarily invade all embryo/placenta pairs following maternal infection. Interestingly, the experiments in Fig 2B suggest that there may be some level of discrepancy between placental and embryonic infection, in that three embryos tested positive for the ZIKV genome, while their respective placentas were negative. Conversely, we also detected one occurrence of ZIKV-positive placenta dissociated from embryonic infection. In the first case, embryonic infection without placental involvement is possible because ZIKV injection at 5.5 dpc preceded placental morphogenesis, so that embryos may have become infected very early through non-placental routes [13,30]. Indeed, TORCH viruses may reach the embryo before and during implantation through multiple ways [30,31] such as: direct infection of early trophoblast progenitors (e.g. see [32]), or through intercellular routes; intrauterine viral infections via ascending infection from the vagina through intact fetal membranes, or after loss of membrane integrity [13,30]. Alternatively, ZIKV RNA levels were simply beyond detection. In the second (single) case, placental infection without embryonic involvement is also conceivable, since the placentas, especially the more mature ones, may well be capable of protecting the conceptus from ZIKV invasion for at least some period of time after maternal infection.

**Fig 4:**
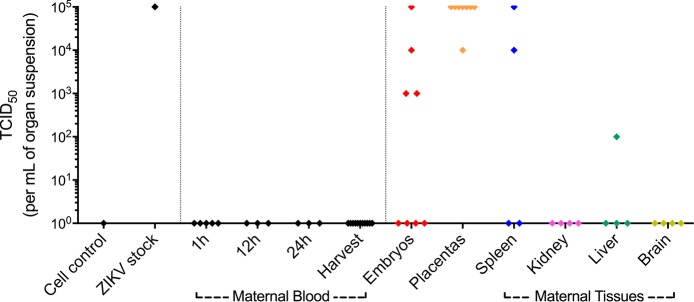
ZIKV infects female organs, placentas and embryos from wild-type immunocompetent mice (FVB/NJ and C57BL/6J), despite the absence of detectable viremia. Maternal blood samples were collected at 1h (n = 5), 12h (n = 3) and 24h (n = 3) after intravascular ZIKV injection at 5.5 days *post-coitum* (dpc), or at 12.5 dpc. Further blood samples, maternal organs, placentas and embryos/fetuses were collected at the day of harvest 10.5 dpc (n = 4) and 11.5 dpc (n = 2), or collected at 16.5 dpc (n = 4) in females injected at 12.5 dpc. Samples were assessed for viral load using a tissue culture infectious dose (TCID_50_) assay in Vero cells. Embryos and respective placentas (n = 8) and maternal organ samples from two 16.5 dpc ZIKV-injected pregnant females (injected in 12.5 dpc), as well as two 12.5 PBS-injected control females were assessed for viral content using a TCID_50_ assay. ZIKV was detected in the spleen of both females and in the liver of one female. Notably, all placentas had significant amounts of ZIKV, whereas just half of the embryos were ZIKV positive. No malformations were observed.

To test the concept that increased placental maturity may constitute a significant physiological barrier to the spread of viable ZIKV particles from the pregnant female to the conceptus, we set out to determine viable viral contents using a TCID_50_ assay. On a subset of the animals represented in Fig 4 (n = 4), we injected ZIKV, or PBS, into 12.5 dpc females and collected maternal blood at 1, 12 and 24 hours post-injection. In the day of harvest (16.5 dpc) we removed a further sample of maternal blood, along with maternal organs, conceptuses and placentas.

Consistent with previous experience with intravascular injections of flaviviruses [13], all blood samples investigated were negative for viable ZIKV particles. This indicates that ZIKV is cleared from the circulation in less than one hour after direct intravascular injection, and that immunocompetent mouse females may not display viable ZIKV particles in their blood throughout the rest of their pregnancies. In contrast, we detected viable ZIKV particles in spleens and livers, while kidneys and brains were negative. This confirms that despite the absence of viremia, ZIKV may reside in a viable form in the spleens and livers of some, but not all immunocompetent females. Importantly, although we detected the highest viral levels in all the placentas, we identified the presence of viable ZIKV particles in only half of the embryos. This indicates that 12.5 dpc placentas are capable of defending the conceptuses from ZIKV in approximately 50% of the cases, consistent with their well-established protective functions against viral threats. We did not find morphological abnormalities in conceptuses from this dataset. As expected, all organs, placentas and conceptuses from control (PBS-injected) females were negative for ZIKV.

### ZIKV phenotypes vary with exposure times and developmental stages

#### Injection at 5.5 dpc, harvest at 10.5 dpc

**Fig 5:**
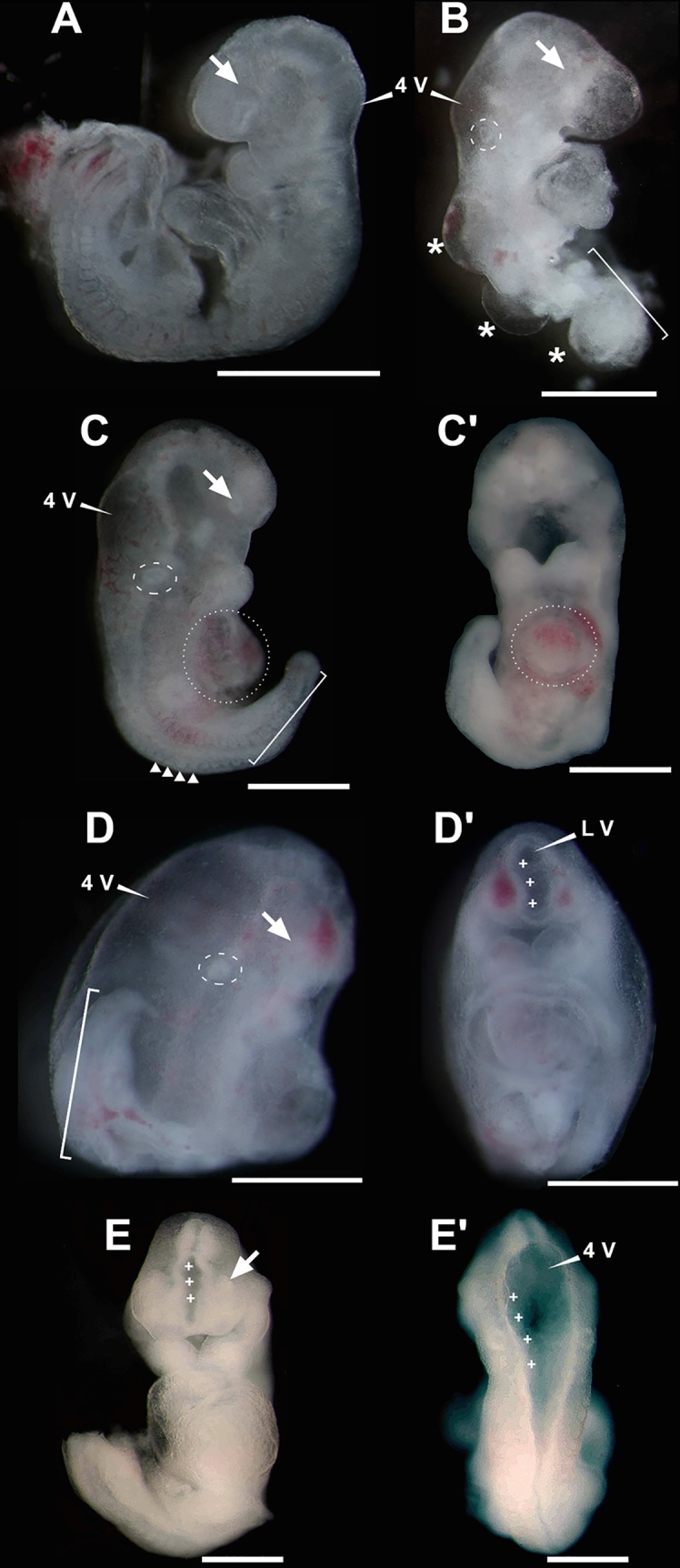
Fetuses from two FVB/NJ pregnant females injected with ZIKV at 5.5 days *post coitum* (dpc) and harvested at 10.5 dpc. (**A-D**) All fetuses were alive at time of harvest. Lateral view of severely affected embryos. (**C’-D’**) Ventral views of embryos depicted in **C-D**, respectively. (**E**) Ventral view of an affected embryo, which was dead at harvest. (**E’**) Dorsal view of embryo depicted in **E**. Note the increased volume of the fourth ventricle and that the neural tube remains opened far below the otic capsule, at the level of the fifth somite, which is abnormally low for 9.5 dpc embryos. In all images: arrow, optic vesicle; asterisks, dorsal edemas; dashed circle, otic vesicle; dotted circle, cardiac *situs inversus*; square bracket, posterior hypotrophy; triangles in **C**, small, compacted somites; cross, dysraphia; 4V, fourth ventricle; LV, lateral ventricle. Scale bars, 0.5 mm.

In the course of our investigation, it became apparent that the phenotypes observed following exposure of pregnant females to ZIKV at 5.5 dpc are contingent on the developmental stages at the day of harvest (e.g. 10.5; 12.5; 16.5 dpc). Briefly, we observed that morphologic consequences are severe in a subset of embryos examined at day 10.5 dpc. Enlargement of the brain ventricles (e.g. hydrocephalus and/or ventriculomegaly) is the most distinguishing feature of the eight malformed embryos we collected at 10.5 dpc (five were alive at the time of harvest). Hydrocephalus is understood as an abnormal increase in the size of lateral, third, or fourth ventricles, resulting from obstructive (non communicant) reasons. When ventricular dilatation is due to causes other than obstruction (communicant), it is often known as ventriculomegaly. However it is difficult to distinguish these types, so that, in practice, hydrocephaly is used for pronounced ventricular dilatations and ventriculomegaly for mild increases in ventricular size [33–35]. In ZIKV affected embryos, hydrocephalus was severe (e.g Fig 5B–5E) and most often manifested by abnormally large fourth ventricles (e.g. Fig 5D). Often, hydrocephalus was associated with single, or multiple foci of dysraphia, either in the anterior neural tube (Fig 5D’ and 5E), or in the spinal cord (Fig 5B, 5E’), suggesting that hydrocephalus may be secondary to dysraphic processes. In the embryo shown in Fig 5B, there were multiple posterior dysraphic segments in the spinal cord associated with fluid blebbing, which are akin to meningoceles. Affected embryos were lagging one, or two days behind in development, and presented additional important abnormalities such as gross caudal body truncation (Fig 5B–5D), cardiac *situs inversus* (Fig 5C–5C’), hypotrophic eyes (Fig 5A–5E’) and otic placodes(Fig 5B–5D), edema (Fig 5A–5D), as well as frequent blood pooling (Fig 5C–5D’). Lower body truncation was associated with the presence of condensed and small somites (Fig 5C).

#### Injection at 5.5 dpc, harvest at 12.5 dpc

**Fig 6:**
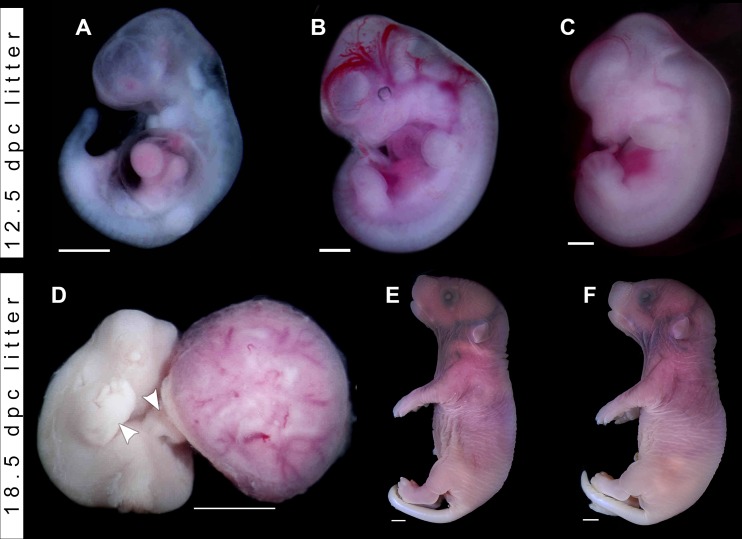
Phenotypes of malformed embryos and fetuses harvested from wild-type pregnant females injected with ZIKV. (**A-B**) Embryos from a 12.5 days *post coitum* (dpc) litter harvested from a C57BL/6J female injected with ZIKV at 5.5 dpc. **A**, Malformed embryo displaying pericardial edema, unfused mandibular arch, missing hyoid arch, abnormal forelimbs and hindlimb rudiments, consistent with developmental delay and growth restriction (staged at 9.5 dpc, or Theiler stage 15). (**B**) Outwardly normal littermate of the embryo depicted in **A** (staged at 11.5 dpc, or Theiler stage 19). (**C**) Control FVB/NJ embryo from a PBS-injected 12.5 dpc pregnant female (staged at 12.5 dpc, or Theiler stage 20–21). (**D**) Malformed fetus from an 18.5 dpc litter harvested from a C57BL/6J female injected with ZIKV at 7.5 dpc. Note the abnormal postures of the right fore and left hind limbs, which are reminiscent of arthrogryposis in humans (white arrowheads), with splayed out forelimb and hindlimb digits, typical of 14.5 dpc (Theiler stage 22). In contrast, eye and ear landmarks are compatible with 11.5 dpc (Theiler stage 19), suggesting that the conceptus was already developmentally delayed at the time of its death (see S2, S5 Tables). The malformed conceptus is displayed along with the maternal side of the placenta. (**E**) Outwardly normal littermate of the fetus in **D**. The conceptuses were staged at 18.5 dpc (Theiler stages 26–27). (**F**) Control C57BL/6J fetuses from a pregnant female injected with PBS at 7.5 dpc and harvested at 18.5 dpc. The fetuses were staged at 18.5 dpc (Theiler stages 26–27). Scale bars, 1.0 mm.

In Fig 6A we show that, contrary to what has been reported [11], C57BL/6J females also produce malformed embryos when exposed to ZIKV. In these females ZIKV was injected at 5.5 dpc and embryos were harvested at 12.5 dpc. In contrast to its apparently normal littermates (Fig 6B), and to embryos harvested from PBS-injected pregnant females (Fig 6C), the affected C57BL/6J embryo presented a pronounced pericardial edema and was dead at harvest (Fig 6A). Judging from embryonic landmarks such as fore and hind limb buds, and from the relative positions of cardiac chambers [20], the embryo presumably died at about 9.5 dpc. As expected, the affected embryo failed to display all the landmarks of the 9.5 dpc stage (Theiler 15) (e.g. note the absence of the mandibular arch), suggesting that it was already trailing behind in development (Fig 6A, S4 Table). Thus, although the malformed embryo was dead, the extent of morphogenetic damage was less than that observed in malformed embryos exposed at 5.5. dpc and harvested at 10.5 dpc. None of the control embryos from PBS-injected mothers displayed any of the above-mentioned alterations (Fig 6C).

#### Injection at 7.5 dpc, harvest at 18.5 dpc

In Fig 6D–6F, we show the results of ZIKV disruption in an 18.5 dpc litter of a C57BL/6J pregnant female infected with ZIKV at 7.5 dpc. The litter contained one abnormal embryo and seven apparently normal littermates. The damaged fetus (Fig 6D) showed signs of necrosis, but its morphology was sufficiently preserved to allow the scoring of typical landmarks associated with specific developmental days. The affected fetus displayed major limb features consistent with developmental day 14.5, but other minor characteristics were compatible with embryonic day 11.5 [20]. It is important to note that this embryo displayed abnormal fore and hindlimb postures, which are analogous to arthrogryposis in human conceptuses infected with ZIKV [36]. As with other abnormal siblings from ZIKV-infected dams, the affected individual was already lagging behind when it died (S5 Table).

#### Injection at 9.5 dpc, harvest at 16.5 dpc

**Fig 7:**
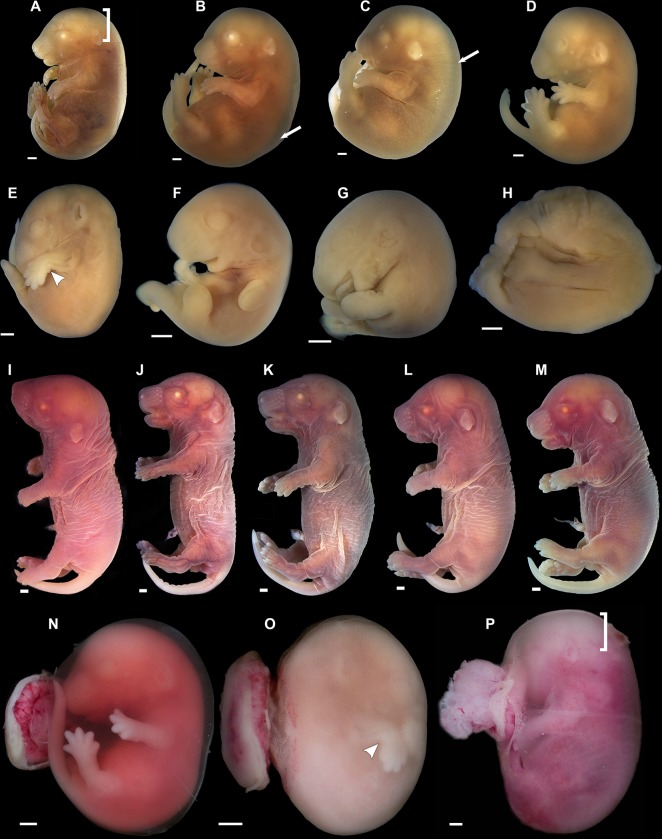
Fetuses from FVB/NJ pregnant female injected with ZIKV at 9.5 days *post coitum* (dpc). Fetuses were harvested at 16.5 dpc. (**A-H**) Fetuses are displayed in an order of increased severity of developmental malformations. (**I-M**) Outwardly normal littermates. (**N-P**) Fetal phenotypes recorded immediately after harvesting and initial dissection. Prominent features of malformed embryos ranged from vascular rarefaction and pallor in the cephalic region (**A**, **P**) (square bracket), generalized edema (**B**, **C**) (arrows) to complete disruption of normal development (**E**-**H**). Note the intra amniotic hemorrhage in **N**, clouding of the amniotic fluid in **O** and the preferential pallor and/or vascular rarefaction in the cephalic region in **P** (square bracket). White arrowhead indicates abnormal forelimb postures suggestive of arthrogryposis. Scale bar, 2.0 mm.

Fig 7A–7H displays eight malformed FVB/NJ conceptuses produced by maternal ZIKV injection at 9.5 dpc, along with five normally shaped littermates (i.e. eight out of thirteen conceptuses display malformations) (Fig 7I–7M). It is readily apparent that, although malformed, these individuals display a substantial degree of correct patterning and morphogenesis. All malformed embryos were dead at the time of harvest (16.5 dpc). In the malformed individuals, the body was typically whitish, and showed signs of lysis, fragmentation and absorption (Fig 7F–7H). ZIKV-induced abnormalities included features as intra-amniotic hemorrhage, clouding of the amniotic fluid (Fig 7N and 7O) and evidence of severe fetal distress, such as local and generalized edema (arrows, Fig 7B and 7C), as well as abnormal articular postures reminiscent of arthrogryposis, as described in humans [36] (Fig 7E and 7O). Importantly, there were signs of vascular rarefaction, more prominent in the cephalic region (brackets, Fig 7A and 7P). The most severe phenotypes were associated with pallor and vascular rarefaction throughout the fetal body (Fig 7B–7H). As judged by their developmental landmarks, affected conceptuses were already dead for one to four days before harvest (16.5 dpc), i.e., they presumably died at embryonic days 12.5 to 15.5 dpc (S2 and S3 Tables).

### In the majority of cases ZIKV exposure reduces cephalic parameters in proportion to body size

**Fig 8:**
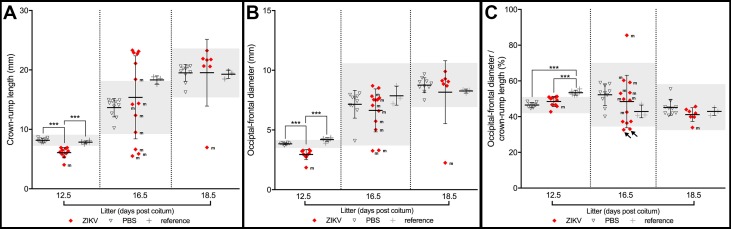
Morphometric analyses in three litters of ZIKV-injected pregnant mouse females. (**A**) Crown rump length (CRL). (**B**) Occipital-frontal diameter (OFD). (**C**) Cephalic proportion relative to body size as indicated by the ratio between OFD/CRL. All data are displayed in relation to an interval defined as the average ± three standard deviations from respective PBS-injected controls. Arrows in **C** indicate two conceptuses displaying marginal evidence for microcephaly. m, malformed conceptuses, ***p<0.001.

**Fig 9:**
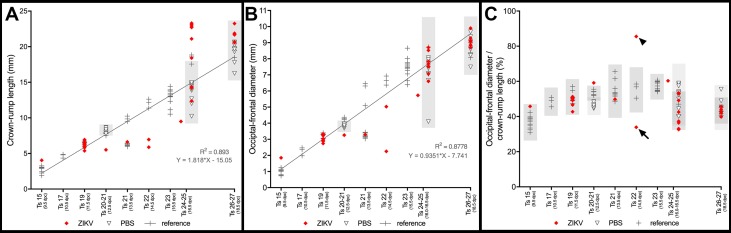
Morphometric analyses in three litters of ZIKV-injected pregnant mouse females. (**A-C**) To better normalize the morphometric results we plotted Crown rump length (CRL) and Occipital-frontal diameter (OFD) as a function of individual embryonic/fetal stages, rather than as nominal litter stages. All conceptuses were classified according to Kauffman [20] and Theiler [22]. (**A**) CRL data. (**B**) OFD data. (**C**) OFD/CRL data. All data are displayed in relation to intervals defined as the average ± three standard deviations of PBS-injected (light grey), or reference controls (dark grey). Stage normalization eliminated all evidence for specific changes in cephalic proportions, but for two conceptuses. One fetus (shown in Fig 7H) displayed an abnormally increased OFD/CRL, which resulted from cephalic collapse in the cranio-caudal axis (arrowhead), while the other fetus (shown in Fig 6D) constitutes the only specific evidence for microcephaly in our study (arrow).

A substantial number of embryos/fetuses exposed to ZIKV at 5.5, 7.5 or 9.5 dpc and harvested at 12.5, 16.5 or 18.5 dpc (Fig 6A and 6D and in Fig 7A–7H; labelled with an “m” in Fig 8B) displayed small head dimensions as indicated by the occipito-frontal diameter (OFD). These malformed embryos/fetuses (9 out of 10) failed to display the complete set of developmental landmarks associated with their estimated ontogenetic stages at the time of death (S2–S5 Tables) [20,22] and were typically small in relation to their phenotypically normal littermates, to embryos from PBS-injected dams, or to non-injected reference conceptuses from FVB animals of our mouse facility. Even if these ZIKV-exposed conceptuses display small OFDs, in the most rigorous classification the diagnosis of microcephaly is only established when cephalic parameters are three standard deviations (SD) below correctly age and stage-matched controls [37]. Thus, when we compared normalized OFDs from all embryos/fetuses associated with ZIKV-injected dams with the interval defined by three SDs, only two fetuses displayed evidence of microcephaly (Fig 8C, arrows). Interestingly, none of these two fetuses showed obvious morphological abnormalities. Importantly, when we staged embryos/fetuses according to established ontogenetic landmarks, rather than by the nominal day of litter development, we determined that cephalic proportions were within the interval defined by three SDs at each specific stage in all but two malformed conceptuses (Fig 9A–9C). One fetus, depicted in Fig 6D constituted the only objective evidence for microcephaly in our study. Interestingly, the other anomalous, grossly malformed fetus (Fig 7H) displayed a disproportionally increased OFD, secondary to collapse of the cephalic region in the cranio-caudal axis (arrowhead Fig 9C).

It is important to note that PBS-injected females did not show any consistent reduction in litter size in relation to our control, uninjected, reference litters. This indicates that all injected females tolerated well the experimental stresses associated with anesthesia and surgical procedures without detrimental effects on intrauterine growth.

### Placental damage associated with exposure to ZIKV

It has been reported that ZIKV infection in humans associates with fetal damage to only one fraternal twin in a sib pair [17]. This anecdotal finding suggests that there are significant physiological and/or immunological checks to embryonic/fetal infection, even after ZIKV gains the circulation. Consistent with this, five out of thirteen (5/13) littermates from FVB/NJ dams injected with ZIKV at 9.5 dpc were outwardly normal (Fig 7, Table 1). Likewise, six out of eight conceptuses (6/8) from dams injected with ZIKV at 5.5 dpc, or eight out of nine fetuses (8/9) from C57BL/6J pregnant females injected with ZIKV at 7.5 dpc were apparently unaffected on purely morphological grounds.

One of the likely physiological checks to ZIKV infection in a conceptus is the placenta. In a successful pregnancy, the placenta plays a crucial role in enabling vital exchanges between mother and conceptuses, as well as in protecting them from microorganisms [38]. Therefore, we hypothesized that the placenta may play a significant role in the pathophysiology of ZIKV-induced embryonic/fetal disruption.

**Fig 10:**
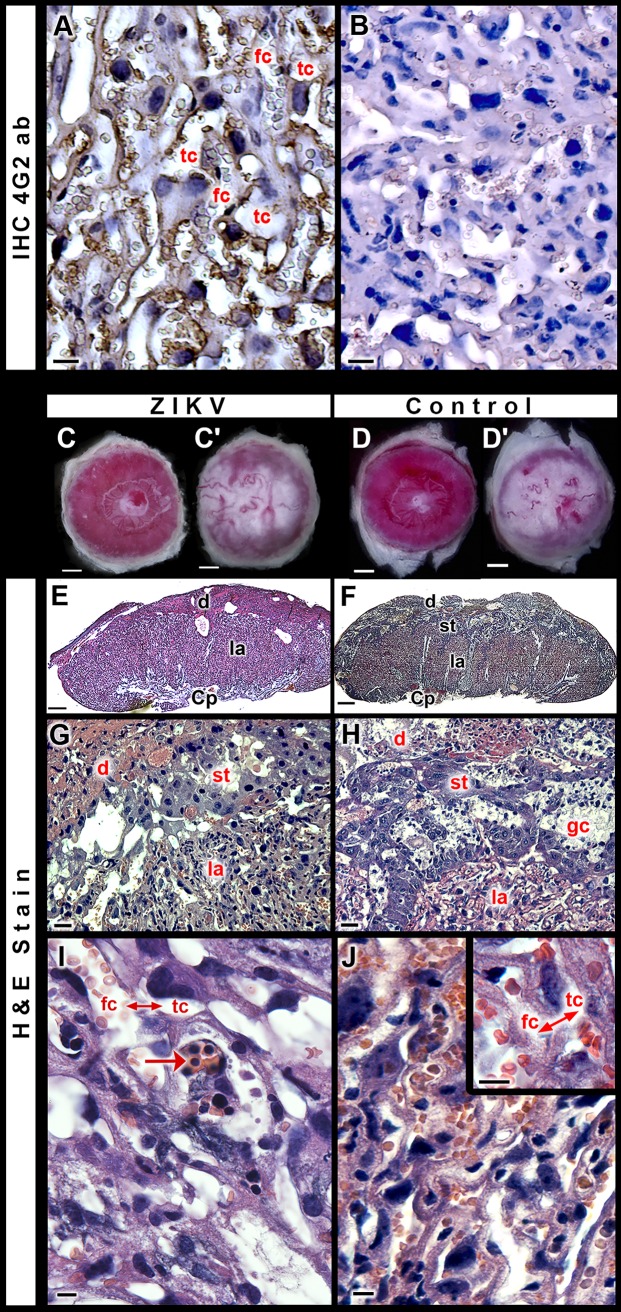
Placental damage associated with exposure to ZIKV. (**A**) Immunoperoxidase reactivity for the flaviviral E protein utilizing the 4G2 monoclonal antibody counterstained with Mayer's Hematoxylin in the labyrinth of a placenta from an outwardly normal fetus from a pregnant females exposed to ZIKV at 9.5 and harvested at 16.5 dpc. Scale bar, 20 μm. Placentas from embryos without structural abnormalities (**A**) show immunoperoxidase reactivity for ZIKV (brown) in the labyrinthine area mainly in the coating of trophoblast cells in the channels filled by maternal blood (tc). (**B**) Negative control of the immunohistochemical reaction. Placentas were harvested from conceptuses of a 16.5 dpc ZIKV-exposed litter and from a non-injected counterpart. (**C-D'**) Macroscopic views of placentas from: an outwardly normal, ZIKV exposed, fetus (**C-C'**); a control fetus from a PBS-injected dam (**D-D'**). **C-D**, fetal side; **C'-D'**, maternal side. Scale bars **C-D'**, 1 mm. (**E-F**) Panoramic views of the histological structure in placentas from an outwardly normal, ZIKV exposed, fetus (**E**) and a control fetus from a PBS-injected dam (**F**). Scale bars, 400 μm. (**G, H**) A higher magnification view of the placentas respectively displayed in **E, F**. Scale bars, 50 μm. Panels (**G**) to (**H**) show the major placental layers: decidua (d), junctional zona with spongiotrophoblast (st), and labyrinth (la). (**I-J**) A labyrinthine view. Placentas from an outwardly normal, ZIKV exposed, fetus (**I**) and a control fetus from a PBS-injected dam (**J**). Scale bars, 20 μm. The inset in (**J**) depicts the interhemal membrane (↔) between fetal capillaries (fc) and trophoblast channels (tc) filled with maternal blood. Nucleated red blood cells (arrow in **I**) are seen in all ZIKV infected placentas. Scale bar, 20 μm. d, decidua; Cp, chorionic plate; H & E, Hematoxylin and Eosin stain, st, spongiotrophoblast; fc, fetal capillary.

In Fig 10, we provide evidence that ZIKV-induced abnormalities correlate with a severe pattern of placental injury in malformed embryos harvested from pregnant females exposed to the virus at 5.5–9.5 dpc. In contrast, exposure to ZIKV outside of the window of susceptibility to malformations (e.g. at 12.5 dpc) was associated with infected placentas, but overtly normal fetuses. This suggests that increased placental maturity can afford some level of protection against ZIKV-induced teratogenesis.

To establish whether placental alterations are directly associated with ZIKV, we performed immunohistochemistry with the anti-flavivirus 4G2 antibody. Fig 10A indicates that placentas from outwardly normal ZIKV exposed fetuses stain positive for the flaviviral antigen, which is consistent with our qRT-PCR and Western blot data (Fig 2E, Fig 3A amd 3B). The staining is concentrated in the labyrinthine area, which is the site where metabolic exchanges between maternal and fetal organisms take place [38]. Trophoblast channel walls are often compromised (Fig 10A, negative control shown in Fig 10B), while the labyrinths from control placentas do not show any ZIKV staining.

At closer inspection, 16.5 dpc placentas from ZIKV disrupted embryos/fetuses showed fibrosis, resorption and hemorrhagic areas of varying dimensions, as compared to placentas from outwardly normal ZIKV exposed fetuses, or to placentas from reference conceptuses (Fig 10C –10F). As before, the most relevant alterations were recorded in the labyrinthine layer (Fig 10G–10H). Placentas from outwardly normal littermates were histologically similar to control placentas, but exhibited persistence of nucleated red blood cells in the labyrinthine fetal capillaries (Fig 10I). This suggests a delay in erythroid differentiation and/or a physiological response to fetal hypoxia.

**Fig 11:**
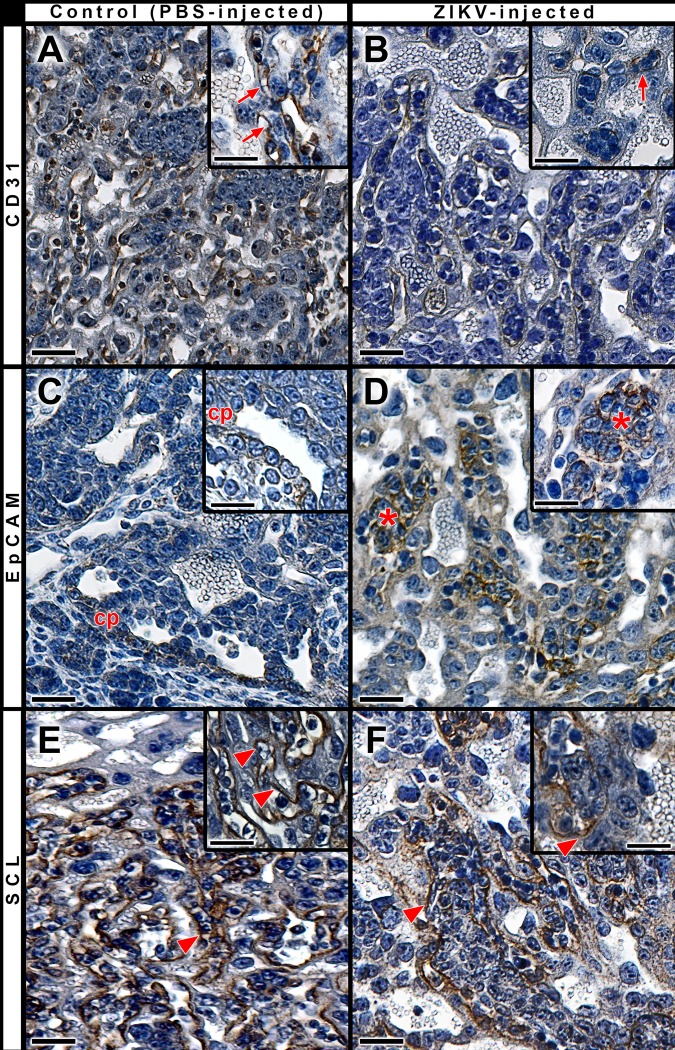
Early placental changes associated with exposure to ZIKV. Immunoperoxidase reactivity (brown) for CD31, EpCAM and SCL in labyrinthine placental area. Sections were counterstained with Mayer's hematoxylin. Pregnant females were PBS-injected (**A, C, E**) or ZIKV-exposed (**B, D, F**) on 5.5 dpc and the placentas were harvested on 12.5 dpc. At the maternal-fetal barrier, there was a less intense staining for CD31 and for SCL in ZIKV-infected mothers (**B, F**) in comparison with PBS-injected controls (**A** and **E**, respectively). A higher-magnification view of the placentas respectively show staining on endothelial cells (arrows, inserts in **A** and **B**) and syncytial layers (arrowheads, inserts in **E** and **F**). Immunoreactivity to EpCAM is restricted to the chorionic plate (cp) in control placenta (**C** and insert) and in cell agglomerates (*) throughout the labyrinth area of placentas exposed to ZIKV (**D** and insert). Scale bars, 100 μm and 50 μm in inserts.

Placentas collected at 12.5 dpc of pregnant females infected with ZIKV, or injected with PBS on 5.5 dpc showed all the patterned placental layers: chorionic plaque, labyrinth and junctional zone. However, when compared to control placentas, placentas of infected mothers exhibited interhemal membranes separated by large areas filled with cellular clusters. Immunoreactivity to CD31, which localizes endothelial cells, underscored this abnormal feature (Fig 11A and 11B). The cell clusters that abounded in the labyrinth of these placentas were stained by EpCAM (Fig 11C and 11D), a marker of multipotent labyrinth trophoblast progenitor cells [39]. It is likely that functional alterations are associated to these morphologic changes. Indeed, the expression of the monocarboxylate transporter SCL16A3, essential for the transport of lactate, ketone bodies and other monocarboxylates [40], also showed a weaker and less extensive staining throughout the placental barrier in infected, rather than in control placentas (Fig 11E and 11F). Collectively, these data suggest a critical developmental change in the ZIKV-challenged mouse placenta, hinting at a disequilibrium between endothelial function and precursor expansion, which may compromise both the exchange and barrier functions of the placenta.

## Discussion

Here we report a mouse model of ZIKV-induced embryonic and fetal disruption via a hematogenic route. Of special significance, our model employs immunocompetent mice of two widely available strains, FVB/NJ and C57BL/6J, and is the first to report the early hydrocephalus that precedes microcephaly in humans [5], as well as the typical affected postures analogous to arthrogryposis in ZIKV-infected humans [36]. Conveniently, the model does not require special resources such as genetically-modified, immunocompromised animals, or expensive procedures such as injections of blocking antibodies. These features provide a better parallel with the human condition [36] than previous reports of ZIKV infection in genetically-modified animals [14,15,26,41], or in *in vitro* brain organoids [42], which cannot model the dynamic between mother and conceptus. Importantly, our approach reproduces many of the biological challenges faced by the virus after it gains access to the blood stream. This aspect contrasts with other possible approaches, in which the virus is injected into the brain [43], or may gain artificial access to the embryo through direct injections into the amniotic fluid. Our model requires surgical manipulation and laboratory skills obtained in most medical and veterinary research environments. This is an important aspect of our approach, because it will contribute to increase the scientific base of research on ZIKV teratogeny.

### ZIKV-induced morphogenetic and maturation defects

In our model, ZIKV affects development at initial phases such as neurulation and beyond. In general, early exposure is associated with important morphological defects, while late exposure is often associated with IUGR, rather than with overt anatomical consequences.

When embryos are exposed to ZIKV within the 5.5-to-9.5 dpc window of susceptibility (akin to the second and third weeks in humans), the resulting phenotypes are varied. Nonetheless, it is already possible to outline a clear and logic succession of morphologic consequences according to the developmental day of exposure and of harvest. An example of the increased morphogenetic severity associated with early harvests can be found, for example, in a comparison between two datasets of embryos exposed at 5.5 dpc, but harvested at 10.5 dpc, or at 12.5 dpc. It is possible to conclude that the most dramatic, devastating, phenotypes are observed at 10.5 dpc (Fig 5), which include dysraphia of the anterior (telencephalon/rhomboencephalon) and posterior neural tube (spinal cord), hydrocephalus, posterior truncation, as well as stunted development of eyes and ears. In contrast, at 12.5 dpc, we detected posterior, mostly caudal, hypotrophy, as well as hypotrophic eyes (apparently arrested at an early optic pit stage) and otic placodes (Fig 6A). Although most of the 10.5 dpc embryos exposed to ZIKV at 5.5 dpc were alive (judging from their beating hearts), the defects associated with them were so overwhelming that one would not expect to find living embryos at 12.5 dpc. In fact, the affected embryo exposed at 5.5 dpc and harvested at 12.5 dpc was dead. Consistent with this interpretation, the number of dead embryos undergoing reabsorption does increase according to the day of harvest in embryos exposed at 5.5 dpc, going from zero at 9.5 dpc, one at 10.5 dpc, four at 11.5 dpc and five at 13.5 to 15.5 dpc (Table 1).

The importance of the embryonic day of exposure to the gravity of ZIKV-induced phenotypes is almost self-evident when we compare the morphological outcomes of embryos exposed at 5.5 dpc to those exposed at 9.5 dpc (Fig 5 and Fig 7, respectively). The morphologies of the former group reflect the dire consequences of interference with early, basic embryonic morphogenesis. In contrast, in the latter group the defects (e.g. amniotic hemorrhage, generalized edema, blood pooling, posterior hyperemia, anterior pallor and vascular rarefaction), while lethal, are clearly associated with interference with maturation, rather than with morphogenetic processes, which further underscores the stepwise reduction in severity with developmental progression.

In summary, we believe that the paradigm of more frequent early morphogenetic consequences and more pronounced late disturbances in maturation suggested by our results will be a useful guide to understand the complex pathogeny of ZIKV-induced damage to the human conceptus.

### ZIKV-associated neural tube defects in mice and their relevance to the human condition

Even from the standpoint of our limited series of neural tube defects associated with embryonic exposure to ZIKV, it is apparent that there are two major components within the brain and spinal cord phenotypes that we described, namely: dysraphia and hydrocephalus. As we show in Fig 5B, 5D, 5D’, 5E and 5E’, dysraphia and hydrocephalus coexist in affected embryos. Complete description of neural phenotypes and establishment of cause and effect relationships between dysraphia and hydrocephalus are out of the scope of this initial characterization. However, established clinical and experimental knowledge suggest that these two manifestations are related, and that dysraphia is, perhaps, the more general condition, while hydrocephalus is a consequence [44]. These two phenotypes are reminiscent of complex and varied conditions, such as pre-natal Arnold-Chiari and Dandy-Walker syndromes, or, conceivably, are related to the incipient embryonic stages of these syndromes. These features are rarely reported in human embryos [45], perhaps due to practical problems in the access to very early conceptuses. Consistent with this interpretation, Arnold-Chiari syndrome type two includes hydrocephalus of the fourth ventricle and is associated with myelomeningocele, which is a dysraphic disorder (see Fig 5B). Moreover, the Dandy-Walker syndrome is defined by hypoplasia of the cerebellar vermis and by cystic dilatation of the fourth ventricle, and is sometimes associated with hydrocephaly of the fourth ventricle and rostral ventricles as well as occipital encephalocele, the latter being another form of dysraphia [46]. Many of these anatomic abnormalities have been described in ZIKV exposed and infected human conceptuses [5,36], as well as in TORCH phenotypes [47]. These findings suggest that, in spite of specific characteristics of each disease, there are some stereotypic pathways of aggression and morphogenetic responses and anatomical consequences in humans and in mice. Ultimately, these common features may hold the clues to understand the sequence of events that leads to the drastic neural phenotypes associated with ZIKV infection in the conceptus.

### Comparisons with previous mouse models

Recently, relevant articles described mouse models of ZIKV infection. Four studies reported alternative models of mouse teratogeny by ZIKV [9–11,13], and another work described a model of ZIKV vertical transmission [12]. The models described have their pros and cons. Cugola *et al*. [11] provide high quality data on brain organoid infection by ZIKV. However, the results from Cugola *et al*. [11] contain few specific morphological consequences in fetuses from pregnant females injected with ZIKV. Perhaps this can be attributed to the late timing of injection (13.5 dpc) and/or to the quality of intravascular delivery via caudal vein, which is technically demanding and often leads to animal stress and to subcutaneous extravasation of injected contents [48].

The SJL mutant strain utilized by Cugola *et al*. [11] is known for displaying high levels of circulating T cells and for its propensity to develop experimental autoimmune encephalomyelitis [49–52]. It is currently unclear how increased levels of circulating T-cells in SJL mice would lead to an increased propensity to develop ZIKV-induced damage to maternal and embryonic/fetal tissues as compared to wild-type C57BL/6 mice. Wu *et al*. [12], Yockey *et al*. [13] and ourselves (Fig 1, Fig 2, Fig 3) demonstrated that ZIKV can infect maternal, placental and embryonic/fetal tissues in wild-type C57BL/6 mice. Thus, it is unlikely that there is any fundamental need for increases in T-cell levels for ZIKV teratogeny. In the best scenario, even if there are marginal differences in ZIKV susceptibility among mouse strains due to T lymphocyte function, it is difficult to assume that they represent an important component of the variation in the human setting.

The work of Miner *et al*. [9] is both comprehensive and convincing. However, because of its reliance on a precondition of immunodeficiency (i.e. *Ifnar1* knockout mice), more work is necessary to show whether it will accurately reproduce the human condition. Notwithstanding, the work of Miner *et al*. [9] represents a highly credible demonstration of the complex interplay of factors associated with ZIKV transmission and damage to the conceptus.

It is more difficult to judge the applicability of the results produced by direct brain microinjection of Li *et al*. [10], Wu *et al*. [12] and Shao *et al*. [16] as models for the human disease. This is because the strategy chosen by the authors is more reminiscent of the protocols utilized to grow and passage the virus [43], than to model the relevant pathophysiological steps and checks involved in the expression of the human condition. In our opinion, a better and more realistic balance is provided by approaches such as intraperitoneal, or intravascular injections in wild-type animals, as described by Wu *et al*. [12] and by the present study. Nonetheless, the approach described by Li *et al*. [10] and Wu *et al*. [12] and colleagues may be especially interesting in defining the different susceptibilities among neurons and neural progenitors, once ZIKV eventually finds its way into the central nervous system of the conceptus.

Another aspect that is worth discussing is the ZIKV load administered to pregnant mice. The number of ZIKV plaque-forming units given to mice in Cugola *et al*. [11] reached as far as 2.0 x 10^11^ and, as such, was several orders of magnitude higher than in Miner *et al*. [9], in Wu *et al*. [12], or in the present work, (10^5^ pfu). It is unclear whether the viral challenge utilized by Cugola *et al*. [11] will find any correspondence with the human setting. Nonetheless, although their viral numbers seem elevated, close inspection of the results obtained by Miner *et al*. [9] suggests that the actual viral load administered may be less important than the immune state of the animal. Even if the 10^3^ pfu load administered subcutaneously in *Ifnar1* knockout females in Miner *et al*. [9] compares favorably with the 10^5^ pfu that we utilized intravascularly, our mice did not develop any signs or symptoms of brain infection displayed by the animals in Miner *et al*. [9]. These data suggest that the key parameter involved in the expression of damage to the conceptuses is the interplay between viral load and susceptibility to the virus.

The developmental delay associated with late ZIKV exposure constitutes a vexing problem when the objective is to make direct comparisons between exposed and control conceptuses, because it demands careful assessment of developmental stages before meaningful comparisons can be made. Concerns with the heterogeneity of development are not restricted to embryos/fetuses, but are of paramount importance when comparing the effects of ZIKV in brain organoids. In contrast to the approaches in organoids, our *in vivo* model with *bona fide* embryos and fetuses display a host of morphological characteristics that can be accessed to establish developmental stages. We believe these characteristics make the *in vivo* model a better approximation to the human setting, while brain organoids may be more adequate for high content approaches.

Contrary to what has been reported, here we demonstrate that ZIKV produces severe developmental phenotypes in immunocompetent, wild-type, embryonic/fetal mice. This indicates that, although strain-specific differences in sensitivity to ZIKV may exist, it is less clear whether the levels of type I/II interferon in C57BL/6J constitute an absolute deterrent to maternal-fetal transmission of ZIKV through the placenta [11].

### The pros and cons of the model

Our objective with this contribution was to describe the critical periods and windows of opportunity associated with the complex effects of ZIKV on embryonic and fetal development, rather than to develop a platform for mass studies on therapeutics. For that we reasoned that the best and most original approach was to establish an immunocompetent model, rather than an artificially enhanced method with immunodeficient animals produced through loss of function paradigms, which increase penetrance, but run the risk of biasing results. Notwithstanding the close similarity of our frequencies with the reported low prevalence of congenital defects associated with ZIKV in the human setting (in the report of Schuler-Faccini et al. [36], arthrogryposis is present in only 11% of the individuals) the defects we report are admittedly relatively infrequent (e.g. arthrogryposis). However, these frequencies are also the result of the need to rapid survey multiple developmental stages of exposure and of analysis within an ethical number of experimental animals at each stage. For example, in our data we detected only two instances of arthrogryposis in two separate litters from females exposed and harvested, respectively at days 7.5/18.5 (Fig 6D) and 9.5/16.5 (Fig 7E and 7O). This is in part because a substantial number of embryos was exposed too late for these morphologic defects, or were analysed at stages before morphogenesis of fore and hindlimbs, when arthrogryposis could not be scored. Moreover, the true prevalence of arthrogryposis in our models must be higher than we report due to ZIKV-induced growth restriction, which reduces the number of embryos at suitable stages for diagnosis. At this point our model represents the first demonstration that dysraphia, hydrocephalus and arthrogryposis associated with ZIKV can be modelled and studied in mice. Future use of our model in therapeutic projects will require focus on the appropriate stages of exposure and analysis, as well as in other relevant parameters.

### Concluding remarks

In addition to recent studies, our results suggest the important role played by the placenta in ZIKV embryonic or fetal infection. ZIKV displays multiple characteristics in common with TORCH agents [8,53], notably in that the maternal organism is often asymptomatic, or mildly affected, while the conceptus can be severely compromised [54].

The specific mechanisms that underlie the developmental effects of ZIKV in humans remain to be established. However, we hope that our demonstration that ZIKV produces severe developmental phenotypes such as dysraphia, hydrocephalus, arthrogryposis, placental damage and IUGR in immunocompetent wild-type mice will be useful as a new experimental paradigm to advance research on ways to counter the ZIKV threat.

## Supporting information

S1 Table Examined embryos in morphometric analyses. (DOCX)

S2 Table Staging criteria used for morphological characterization of embryos and fetuses. Adapted from [20].
(DOCX)

S3 Table Morphological characterization and staging of embryos and fetuses from ZIKV-injected pregnant females (16.5dpc). Adapted from [20].
(DOCX)

S4 Table Morphological characterization and staging of embryos from ZIKV-injected pregnant females (12.5 dpc). Adapted from [20].
(DOCX)

S5 Table Morphological characterization and staging of embryos and fetuses from ZIKV-injected pregnant females (18.5dpc). Adapted from [20].
(DOCX)
