# Supplementary material for: Hydrocephalus and arthrogryposis in an immunocompetent mouse model of ZIKA teratogeny: A developmental study
Source: PLoS Negl Trop Dis. 2017 Feb 23;11(2):e0005363. doi: 10.1371/journal.pntd.0005363 (PMC5322881; doi:10.1371/journal.pntd.0005363)
Supplement: S1 Table — (DOCX) [file pntd.0005363.s001.docx]

**S1 Table. Examined embryos in morphometric analyses.**

| **Mouse Strain** | **Harvested at Embryonic Day** | **Treatment** | **Total of embryos examined** |
| --- | --- | --- | --- |
| FVB Reference | 9.5 | Non-injected. | 11 |
| FVB Reference | 10.5 | Non-injected | 3 |
| FVB Reference | 11.5 | Non-injected | 7 |
| FVB Reference | 12.5 | Non-injected | 6 |
| FVB Reference | 13.5 | Non-injected | 11 |
| FVB Reference | 14.5 | Non-injected | 3 |
| FVB Reference | 15.5 | Non-injected | 12 |
| FVB Reference | 16.5 | Non-injected | 3 |
| FVB Reference | 18.5 | Non-injected | 3 |
| C57 | 12.5 | ZIKV (10^5^ pfu) | 9 |
| C57 | 18.5 | ZIKV(10^5^ pfu) | 8 |
| C57 | 18.5 | PBS | 10 |
| FVB | 12.5 | PBS | 9 |
| FVB | 16.5 | ZIKV(10^5^ pfu) | 13 |
| FVB | 16.5 | PBS | 9 |
