# Supplementary material for: Hydrocephalus and arthrogryposis in an immunocompetent mouse model of ZIKA teratogeny: A developmental study
Source: PLoS Negl Trop Dis. 2017 Feb 23;11(2):e0005363. doi: 10.1371/journal.pntd.0005363 (PMC5322881; doi:10.1371/journal.pntd.0005363)
Supplement: S2 Table — Adapted from [20]. (DOCX) [file pntd.0005363.s002.docx]

**Table S2**. **Staging criteria used for morphological characterization of embryos and fetuses.** Adapted from Kauffman, 1994.

| normal embryos  (DPC) | *forelimbs and hindlimbs* | *eyelid* | *pinna of the ear* | *vibrissae* | *sinus hair follicle* | *skin wrinkles* | *umbilical hernia* |  |
| --- | --- | --- | --- | --- | --- | --- | --- | --- |
| 10.5 | limb buds. Apical ectodermal ridge present in both forelimbs and hindlimbs | absent | absent | absent | absent | absent | present |  |
| 11.5 | paddle-shaped hand and foot plates | absent | absent | absent | absent | absent | present |  |
| 12.5 | polygonal shape handplate with digital interzones and paddle-shape foot plate | absent | rudiment | primordia | absent | absent | present |  |
| 13.5 | webbing present in forelimbs and hindlimbs. Digits splayed out and almost symetrical | present not closed | present | present without follicles | absent | absent | present | |
| 14.5 | discrete digits in handplate, vestigial webbing in footplate. Digits splayed out | present not closed | covers 1/3 of the ear | present without follicles | primordia | absent | present |  |
| 15.5 | digits splayed out. Nail primordia in forelimbs | almost closed | covers 1/2 of the ear | present with unerupted follicles | present | absent | present |  |
| 16.5 | parallel digits in forelimb. Pollex smaller. Nails in forelimbs and hindlimbs | closed | covers all the ear | present with erupted follicles | present | present in neck, trunk | absent |  |
| 17.5 | parallel digits and nails in forelimbs and hindlimbs | closed | covers all the ear | Prominent with erupted follicles | present | Present in neck, trunk and hindlimbs | absent |  |
| 18.5 | parallel digits and nails in forelimbs and hindlimbs | closed | covers all the ear | Prominent with erupted follicles | present | Present and generelized | absent |  |
