# Supplementary material for: Hydrocephalus and arthrogryposis in an immunocompetent mouse model of ZIKA teratogeny: A developmental study
Source: PLoS Negl Trop Dis. 2017 Feb 23;11(2):e0005363. doi: 10.1371/journal.pntd.0005363 (PMC5322881; doi:10.1371/journal.pntd.0005363)
Supplement: S3 Table — Adapted from [20]. (DOCX) [file pntd.0005363.s003.docx]

|  |  | | | | | | | |  | |  |  |
| --- | --- | --- | --- | --- | --- | --- | --- | --- | --- | --- | --- | --- |
| EMBRYOS | ***forelimbs and hindlimbs*** | ***eyelid*** | ***pinna of the ear*** | ***vibrissae*** | ***sinus hair follicle*** | ***skin wrinkles*** | ***umbilical hernia*** | ***number of typical landmarks for the stage*** | ***dead*** | | ***estimated stage***  ***(dpc/Ts)*** | |
| 1 | 16.5 dpc | 16.5 dpc | 16.5 dpc | 16.5 dpc | 16.5 dpc | 16.5 dpc | 16.5 dpc | 7/7 | yes | | 16.5 dpc / Ts24-25 | |
| 2 | 13.5 dpc | 15.5 dpc | 15.5-16.5 dpc | 15.5 dpc | 15.5 dpc | 16.5 dpc | 16.5 dpc | 3/7 | yes | 16.5 dpc / Ts24-25 | | |
| 3 | 11.5 dpc | na | 12.5 dpc | 11.5 dpc | na | na | nd | 2/7 | yes | 12.5 dpc / Ts20-21 | | |
| 4 | 14.5-16.5 dpc | 14.5dpc | 15.5-16.5 dpc | 14.5-15.5dpc | 14.5dpc | 15.5-16.5dpc | 16.5 dpc | 4/7 | yes | 16.5 dpc / Ts24-25 | | |
| 5 | 16.5 dpc | 16.5 dpc | 16.5 dpc | 16.5 dpc | 16.5 dpc | 17.5 dpc | 16.5 dpc | 7/7 | yes | 16.5 dpc / Ts24-25 | | |
| 6 | 16.5 dpc | 16.5 dpc | 16,5 dpc | 16.5 dpc | 16.5 dpc | 17.5 dpc | 16.5 dpc | 7/7 | yes | 16.5 dpc / Ts24-25 | | |
| 7 | 16.5 dpc | 16.5 dpc | 16.5 dpc | 16.5 dpc | 16.5 dpc | 17.5 dpc | 16.5 dpc | 7/7 | yes | 16.5 dpc / Ts24-25 | | |
| 8 | 11.5 dpc | 13.5 dpc | 12.5 dpc | 12.5-13.5dpc | na | na | na | 4/7 | yes | 13.5 dpc / Ts21-22 | | |
| 9 | 16.5 dpc | 16.5 dpc | 16.5 dpc | 16.5 dpc | 16.5 dpc | 17.5 dpc | 16.5 dpc | 7/7 | yes | 16.5 dpc / Ts24-25 | | |
| 10 | 13.5 dpc | 14.5 dpc | 14.5 dpc | 14.5 dpc | 14.5 dpc | 16dpc | nd | 1/7 | yes | 16.0 dpc / Ts23-24 | | |
| 11 | 13.5-14.5dpc | 14.5 dpc | 15.5 dpc | 14.5 dpc | 15.5 dpc | 15.5-16.5dpc | 16.5dpc | 2/7 | yes | 16.5 dpc / Ts24-25 | | |
| 12 | 14.5 dpc | 12.5 dpc | 14.5 dpc | 13.5 dpc | 13.5 dpc | nd | nd | 1/7 | yes | 14.5 dpc / Ts22-23 | | |
| 13 | 16.5 dpc | 16.5 dpc | 16.5 dpc | 16.5 dpc | 16.5 dpc | 17.5 dpc | 16.5 dpc | 7/7 | yes | 16.5 dpc / Ts24-25 | | |

**Table S3**. **Morphological characterization and staging of embryos and fetuses from ZIKV-injected pregnant females (16.5dpc).** Adapted from Kauffman, 1994.

**na: not applicable; nd: not determined**
