# Supplementary material for: Hydrocephalus and arthrogryposis in an immunocompetent mouse model of ZIKA teratogeny: A developmental study
Source: PLoS Negl Trop Dis. 2017 Feb 23;11(2):e0005363. doi: 10.1371/journal.pntd.0005363 (PMC5322881; doi:10.1371/journal.pntd.0005363)
Supplement: S4 Table — Adapted from [20]. (DOCX) [file pntd.0005363.s004.docx]

**Table S4.** **Morphological characterization and staging of embryos from ZIKV-injected pregnant females (12.5 dpc).** Adapted from Kauffman, 1994.

|  |  | | | | | | |  |  |  |
| --- | --- | --- | --- | --- | --- | --- | --- | --- | --- | --- |
| EMBRYOS | ***forelimbs and hindlimbs*** | ***eyelid*** | ***pinna of the ear*** | ***vibrissae*** | ***sinus hair follicle*** | ***skin wrinkles*** | ***umbilical hernia*** | ***number of typical landmarks for the stage*** | ***dead*** | ***estimated stage***  ***(dpc/Ts)*** |
| 1 | 11.5 dpc | 11.5 dpc | 11.5 dpc | 11.5 dpc | 11.5 dpc | 11.5 dpc | 11.5 dpc | 7/7 | no | 11.5 dpc / Ts19-20 |
| 2 | 11.5 dpc | 11.5 dpc | 11.5 dpc | 11.5 dpc | 11.5 dpc | 11.5 dpc | 11.5 dpc | 7/7 | no | 11.5 dpc / Ts19-20 |
| 3 | 11.5 dpc | 11.5 dpc | 11.5 dpc | 11.5 dpc | 11.5 dpc | 11.5 dpc | 11.5 dpc | 7/7 | no | 11.5 dpc / Ts19-20 |
| 4 | 11.5 dpc | 11.5 dpc | 11.5 dpc | 11.5 dpc | 11.5 dpc | 11.5 dpc | 11.5 dpc | 7/7 | no | 11.5 dpc / Ts19-20 |
| 5 | 11.5 dpc | 11.5 dpc | 11.5 dpc | 11.5 dpc | 11.5 dpc | 11.5 dpc | 11.5 dpc | 7/7 | no | 11.5 dpc / Ts19-20 |
| 6 | 11.5 dpc | 11.5 dpc | 11.5 dpc | 11.5 dpc | 11.5 dpc | 11.5 dpc | 11.5 dpc | 7/7 | no | 11.5 dpc / Ts19-20 |
| 7 | 11.5 dpc | 11.5 dpc | 11.5 dpc | 11.5 dpc | 11.5 dpc | 11.5 dpc | 11.5 dpc | 7/7 | no | 11.5 dpc / Ts19-20 |
| 8 | 11.5 dpc | 11.5 dpc | 11.5 dpc | 11.5 dpc | 11.5 dpc | 11.5 dpc | 11.5 dpc | 7/7 | no | 11.5 dpc / Ts19-20 |
| 9 | 9.5 dpc | na | na | na | na | na | present | 0/7 | yes | 9.5 dpc / Ts14-15 |
| 10 | 11.5 dpc | 11.5 dpc | 11.5 dpc | 11.5 dpc | 11.5 dpc | 11.5 dpc | 11.5 dpc | 7/7 | no | 11.5 dpc / Ts 19-20 |
